# Supplementary material for: Resolving the full spectrum of human genome variation using Linked-Reads
Source: Genome Res. 2019 Apr;29(4):635–45. doi: 10.1101/gr.234443.118 (PMC6442396; doi:10.1101/gr.234443.118)
Supplement: Supplemental Material [file supp_gr.234443.118_Supplemental_longranger-2.2.zip › longranger-2.2.tar.bz2!/lr2.2-public/lib/stan/cmdstan/stan/doc/api/html/structstan_1_1gm_1_1is__no__op__statement__vis-members.html]

Stan: Member List


|  |  |
| --- | --- |
|  | Stan  2.5.0  probability, sampling & optimization |


- Main Page
- Related Pages
- Namespaces
- Classes
- Files

- Class List
- Class Index
- Class Hierarchy
- Class Members

All Classes Namespaces Files Functions Variables Typedefs Enumerations Enumerator Friends Macros Pages

- stan
- gm
- is\_no\_op\_statement\_vis

stan::gm::is\_no\_op\_statement\_vis Member List

This is the complete list of members for stan::gm::is\_no\_op\_statement\_vis, including all inherited members.

|  |  |  |
| --- | --- | --- |
| operator()(const nil &st) const | stan::gm::is\_no\_op\_statement\_vis |  |
| operator()(const assignment &st) const | stan::gm::is\_no\_op\_statement\_vis |  |
| operator()(const sample &st) const | stan::gm::is\_no\_op\_statement\_vis |  |
| operator()(const increment\_log\_prob\_statement &t) const | stan::gm::is\_no\_op\_statement\_vis |  |
| operator()(const expression &st) const | stan::gm::is\_no\_op\_statement\_vis |  |
| operator()(const statements &st) const | stan::gm::is\_no\_op\_statement\_vis |  |
| operator()(const for\_statement &st) const | stan::gm::is\_no\_op\_statement\_vis |  |
| operator()(const conditional\_statement &st) const | stan::gm::is\_no\_op\_statement\_vis |  |
| operator()(const while\_statement &st) const | stan::gm::is\_no\_op\_statement\_vis |  |
| operator()(const print\_statement &st) const | stan::gm::is\_no\_op\_statement\_vis |  |
| operator()(const reject\_statement &st) const | stan::gm::is\_no\_op\_statement\_vis |  |
| operator()(const no\_op\_statement &st) const | stan::gm::is\_no\_op\_statement\_vis |  |
| operator()(const return\_statement &st) const | stan::gm::is\_no\_op\_statement\_vis |  |


---

[ Stan Home Page ]

*© 2011–2014,
Stan Development Team.*
